# *QuickStats: *Percentage* of Children and Adolescents Aged 5–17 Years Who Had Ever Received a Diagnosis of Attention-Deficit/Hyperactivity Disorder, by Sex and Year — United States, 2019–2025

**DOI:** 10.15585/mmwr.mm7542a2

**Published:** 2026-10-29

**Figure:**
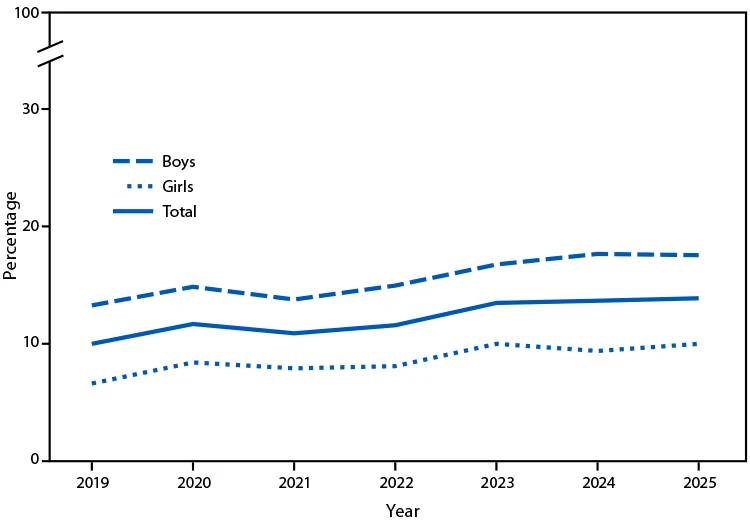
From 2019 to 2025, the percentage of children and adolescents aged 5–17 years who had ever received a diagnosis of ADHD increased from 9.9% to 13.8%. The percentage of boys and girls with ADHD diagnoses increased throughout this period; a higher percentage of boys than girls had ever received a diagnosis of ADHD.

**Supplementary Table:** Percentage of Children and Adolescents Aged 5–17 Years Who Had Ever Received a Diagnosis of Attention-Deficit/Hyperactivity Disorder, by Sex and Year — United States, 2019–2025

**Source:** National Center for Health Statistics, National Health Interview Survey, 2019–2025. About NHIS | National Health Interview Survey | CDC

For more information on this topic, CDC recommends the following link: Attention-Deficit / Hyperactivity Disorder (ADHD) | CDC.

